# Dietary Docosahexaenoic Acid Reduces Oscillatory Wall Shear Stress, Atherosclerosis, and Hypertension, Most Likely Mediated via an IL‐1–Mediated Mechanism

**DOI:** 10.1161/JAHA.118.008757

**Published:** 2018-06-30

**Authors:** Mabruka A. Alfaidi, Janet Chamberlain, Alexander Rothman, David Crossman, Maria‐Cruz Villa‐Uriol, Patrick Hadoke, Junxi Wu, Torsten Schenkel, Paul C. Evans, Sheila E. Francis

**Affiliations:** ^1^ Department of Infection, Immunity and Cardiovascular Disease University of Sheffield United Kingdom; ^2^ INSIGNEO Institute for in silico Medicine & Department of Computer Science University of Sheffield United Kingdom; ^3^ BHF Centre of Excellence University of Edinburgh United Kingdom; ^4^ Department of Engineering and Mathematics Hallam University Sheffield United Kingdom; ^5^ University of St Andrews United Kingdom

**Keywords:** docosahexaenoic acid, endothelium, hypertension, inflammation, interleukin 1, wall shear stress, Atherosclerosis, Animal Models of Human Disease, Basic Science Research, Growth Factors/Cytokines, Hypertension

## Abstract

**Background:**

Hypertension is a complex condition and a common cardiovascular risk factor. Dietary docosahexaenoic acid (DHA) modulates atherosclerosis and hypertension, possibly via an inflammatory mechanism. IL‐1 (interleukin 1) has an established role in atherosclerosis and inflammation, although whether IL‐1 inhibition modulates blood pressure is unclear.

**Methods and Results:**

Male apoE^−/−^ (apolipoprotein E–null) mice were fed either a high fat diet or a high fat diet plus DHA (300 mg/kg per day) for 12 weeks. Blood pressure and cardiac function were assessed, and effects of DHA on wall shear stress and atherosclerosis were determined. DHA supplementation improved left ventricular function, reduced wall shear stress and oscillatory shear at ostia in the descending aorta, and significantly lowered blood pressure compared with controls (119.5±7 versus 159.7±3 mm Hg, *P*<0.001, n=4 per group). Analysis of atheroma following DHA feeding in mice demonstrated a 4‐fold reduction in lesion burden in distal aortas and in brachiocephalic arteries (*P*<0.001, n=12 per group). In addition, DHA treatment selectively decreased plaque endothelial IL‐1β (*P*<0.01).

**Conclusions:**

Our findings revealed that raised blood pressure can be reduced by inhibiting IL‐1 indirectly by administration of DHA in the diet through a mechanism that involves a reduction in wall shear stress and local expression of the proinflammatory cytokine IL‐1β.

**Clinical Perspective**

**What Is New?**

- In a mouse model of atherosclerosis, supplementation of a Western diet with docosahexaenoic acid improved left ventricular function, reduced aberrant hemodynamic flow patterns in the aorta, and lowered blood pressure.
- Atheroma burden was reduced 4‐fold in the docosahexaenoic acid–supplemented group at 2 anatomic sites.
- IL‐1 (interleukin 1) is plausibly implicated in the mechanism for these effects because docosahexaenoic acid selectively decreased plaque endothelial IL‐1β.

**What Are the Clinical Implications?**

- Regular supplementation of the diet with docosahexaenoic acid alters blood‐flow patterns in the vasculature, reducing local IL‐1–induced inflammation and atherosclerosis and, consequently, lowering blood pressure.
- Given the importance of the CANTOS (Canakinumab Antiinflammatory Thrombosis Outcome Study), our data suggest that strategies that control local IL‐1β may also be useful in hypertension.

Hypertension is a major healthcare concern, given its prevalence among the population and its devastating complications, namely, stroke and ischemic heart disease (IHD).^1^ Evidence suggests that hypertension and dyslipidemia are the underlying risk factors in IHD.^2^ In the search for a novel therapeutic intervention, the importance of inflammatory molecules and immune cell involvement in the disease is increasing.

Arterial inflammation has been shown in patients with systemic hypertension.^3^ Cytokines, including IL‐1 (interleukin 1), are key mediators in inflammation.^4^, ^5^ In pathological vascular disease, the main role of IL‐1 is to regulate leukocyte migration and accumulation.^6^, ^7^ IL‐1 has also been shown to promote endothelial cell dysfunction,^8^ a cardinal process in the pathogenesis of hypertension and atherosclerosis. Several studies, in animals^9^, ^10^ and humans,^11^, ^12^ have postulated a key role for IL‐1β in the pathogenesis of atherosclerosis and clinical sequelae such as hypertension.^13^, ^14^

Recent studies suggest that dietary fat, especially saturated fatty acids, are deleterious for atherosclerosis and hypertension.^15^ Although saturated fat is proatherogenic, ω‐3 fatty acids (n3FA), especially docosahexaenoic acid (DHA; 22:6n‐3), are known to be anti‐atherogenic,^16^ with multiple underlying mechanisms.^17^ Consequently, it has been suggested that patients at high risk of the disease need to increase their fish and fish‐derived food intake and decrease saturated fat intake.^18^, ^19^

Epidemiological, case–control, and prospective clinical studies have demonstrated positive effects of n3FAs in the reduction of cardiovascular mortality rates among IHD patients.^20^ These effects have been ascribed to the improvement of cardiovascular risk profiles, resulting in disease prevention^21^; however, the data on IHD and hypertension are less robust. Clinical trials in post–myocardial infarction patients have suggested that DHA, specifically, may exert a therapeutic effect, particularly on hypertension and the risk of reinfarction,^22^ although this has not been shown unequivocally or linked with atherosclerotic plaque stabilization/regression.^23^, ^24^ DHA supplementation decreased blood pressure in spontaneously hypertensive rats^25^, ^26^ and in a dog model of hypertension,^27^ but no data are available for mouse models with atherosclerosis.

Consequently, it is clear that DHA has broad anti‐inflammatory effects, but whether it has inhibitory effects on IL‐1–dependent inflammation in the setting of hypertension and the mechanisms involved needs further elucidation.

We aimed to investigate whether DHA supplementation in mice could alter vascular contractility via an IL‐1β–mediated mechanism, contributing to blood pressure reduction in experimental atherosclerosis. We showed that DHA reduces wall shear stress (WSS; calculated by computational flow dynamics) in the descending aorta, especially at ostia, and that this is associated with significantly decreased arterial blood pressure, enhanced left ventricular (LV) function, and a reduction in intraplaque inflammation directed by IL‐1β.

## Methods

The data, analytic methods, and study materials will not be made available to other researchers for purposes of reproducing the results or replicating the procedure; however, requests for specific elements of the these materials will be considered.

Detailed methods can be found in the supplemental information (Data S1).

### Diet‐Induced Hypertension in Mice

Male ApoE^−/−^ (apolipoprotein E–null) mice were bred in‐house at the University of Sheffield. Food and water were given ad libitum in a controlled environment (temperature 22–25°C, humidity 55*±*5%, and 12‐hour light cycle). Starting at 8 weeks of age, the mice were housed individually and randomly separated into 1 of 2 groups (n=12 per group). The control group was fed a Western‐type high‐fat diet (HFD) containing 21% (wt/wt) fat, 0.15% (wt/wt) cholesterol, and 0.296% (wt/wt) sodium, whereas the DHA‐treated group received the HFD and DHA (99% purified). In this model, mice have raised blood pressure as a result of HFD feeding.^9^

DHA feeding was achieved by mixing the DHA with jelly using a previously described “jelly‐delivery” protocol^28^ at a final concentration of 300 mg/kg per day. Briefly, DHA was placed into separate shallow molds (12 doses of DHA per mold). Flavored jelly was dissolved in hot water, according to the manufacturer's instructions and allowed to cool, then poured into each mold and mixed with the DHA to achieve an even suspension. Molds were placed immediately in a freezer and later cut into 12 equal cubes, measuring ≈1 cm^3^, containing a single dose of drug. The mice were monitored daily for their DHA intake. Body weights were measured weekly over 12 weeks. All animal care and procedures were closely conducted under ASPA 1986, UK and in accordance with Home Office guidelines under license 70/7992.

### Biochemistry

To confirm absorption of DHA, red blood cell ω‐3 composition and indexes were measured by gas chromatography in pooled blood samples of the mice (4 mice per group).^29^

### Blood Pressure Analysis

Systolic and diastolic blood pressure was measured in the mice using a Visitech tail‐cuff system, as described previously.^9^

### Echocardiography

Echocardiograms were performed in the mice fed either a HFD alone or a HFD and DHA over 12 weeks, as described previously.^30^

### Atherosclerosis Analysis

The extent of atherosclerosis was assessed in cross‐sectional aortic sinus and brachiocephalic sections, as described.^31^ Mean lesion size was calculated from measurements of 5 sinus sections starting from the 3 cusp area. Analysis was conducted blinded per individual animal and experimental conditions, and measurements from all sections averaged per mouse. A second assessment of atherosclerosis was conducted in the whole aortas using an en face method.^32^ Lesion areas were analyzed using a NIS‐Elements software system (Nikon).

### Immunohistochemical Analysis

Paraffin‐embedded aortic sections were immunostained, as described previously.^33^ Heat‐mediated antigen retrieval was performed using citrate buffer (pH 6.0). Following blockage of nonspecific binding by incubation with either 1% (wt/vol) bovine serum albumin or 1% (wt/vol) nonfat milk, the sections were incubated with the relevant primary antibody overnight at 4°C (anti–IL‐1β, 1:100, or anti–IL‐1ra, 1:100). Positive staining was detected using a biotinylated secondary antibody followed by an avidin/biotin complex with horse radish peroxidase label (ABC‐HRP complex) and visualized using DAB. Cell nuclei were counterstained using hematoxylin. Images were taken and analyzed using NIS‐Elements software.

### Computational Fluid Dynamics

Computational fluid dynamics of the unsteady flow in the aortic branch, including the ostia, was performed using the unsteady Navier‐Stokes code ANSYS Fluent 17.1, following a similar model to that used by Luong et al.^34^ The geometry of a single representative murine aortic geometry was derived from postmortem optical projection tomography.^35^ Briefly, the thoracic descending aorta was carefully dissected and embedded in 1.5% (wt/vol) low‐melting‐point agarose gel. The sample was dehydrated in pure ethanol (twice, 24 hours each) and optically cleared in BABB (benzyl alcohol and benzyl benzoate at a 1:2 ratio) for 24 hours. The aorta was scanned using a Bioptonics 3001 tomograph (Bioptonics). Projection images (×400) were obtained with a 0.9° rotation step, and these were reconstructed into a 3‐dimensional image that contained a stack of 1024 cross‐sections using NRecon software (Skyscan). The 3‐dimensional image was then triangulated into a high‐resolution STL (standard triangle language) surface, and an intermediate surface mesh comprising 2.2 million triangles was produced using image processing and reconstruction (ImageJ v1.49u^36^) and image segmentation (3‐dimensional slicer v4.5.0^37^) software. Further mesh manipulation and cleaning were performed using Paraview v4.3.1^38^ and the specialized vascular modeling software @neuFuse v7.3 to produce a high‐resolution mesh comprising 1.3 million triangles. This resolution was chosen as a compromise to accommodate the wide range of length scales from the aortic arch down to the ostia diameters required in this study.

The lumen was meshed using ANSYS ICEM computational fluid dynamics, generating a hybrid tetrahedral mesh with a prismatic boundary layer representation. Based on a mesh independence analysis,^34^ a final volume mesh size of 1 million cells was used to deliver converged WSS, with simulation times of 36 hours on 4 cores of an Intel i7 3.4 GHz processor. The geometry was assumed to be rigid. Blood was modeled as a Newtonian fluid with a dynamic viscosity of η=0.004 Pa/s and a density of ρ=1235 kg/m^3^. Calculated with a typical maximum flow speed of V=1 m/s and a hydraulic diameter at the aortic root of D=1.58 mm, the maximum Reynolds number is $\text{Re}=\left(\rho \text{VD}\right)\eta^{-1}=488$. The flow was thus modeled as laminar.

The inlet boundary condition was a time‐dependent prescribed velocity profile (Dirichlet condition), calculated as a third‐order Bezier spline approximation^39^ based on a digitized US Doppler measurement.

Outlet conditions at all exits were von Neumann outlet conditions with a split of 69.8%, 16%, 8%, and 6% for descending aorta, innominate, common carotid, and subclavian, respectively, and 0.14% for each of the 12 ostia. All outlet conditions were in phase with the inlet velocity profile.

Numerical solutions were obtained using cell‐based gradients with second‐order pressure, quadratic upwind momentum, and second‐order implicit time discretization. Time step size was chosen as 1/1000th of the cycle time. Three cycles were simulated to allow for fully developed periodicity, and the last cycle was evaluated.

Three‐dimensional time‐dependent WSS data were exported to calculate the time‐averaged WSS magnitude (TAWSS), the oscillatory shear index (OSI), and transverse WSS (transWSS):

$$\text{TAWSS}=\frac{1}{T}\;\int_{0}^{T}\left|{\vec{\tau }}_{\omega }\right|\;\text{dt}$$

$$\text{OSI}=\frac{1}{2}\left(1-\;\frac{|\int_{0}^{T}{\vec{\tau }}_{\omega }\;\text{dt}|}{\int_{0}^{\;T}\left|\;{\vec{\tau }}_{\omega }\;\right|\text{dt}}\;\right)=\;\frac{1}{2}\left(1-\frac{\left|{\vec{\tau }}_{\text{mean}}\right|}{\text{TAWSS}}\right),\;\text{where}\;{\vec{\tau }}_{\text{mean}}=\frac{1}{T}\;\int_{0}^{T}{\vec{\tau }}_{\omega }\;\text{dt}$$

$$\text{transWss}=\frac{1}{T}\int_{0}^{T}\left|{\vec{\tau }}_{\omega }\cdot \left(\vec{n}\times \frac{\frac{1}{T}\int_{0}^{T}{\vec{\tau }}_{\omega }\text{dt}}{\left|\frac{1}{T}\int_{0}^{T}{\vec{\tau }}_{\omega }\text{dt}\right|}\right)\right|=\frac{1}{T}\int_{0}^{T}\left|{\vec{\tau }}_{\omega }\cdot \left(\vec{n}\times \frac{{\vec{\tau }}_{\text{mean}}}{|{\vec{\tau }}_{\text{mean}}|}\right)\right|$$

Although OSI quantifies the oscillatory character of near wall flow, transWSS is a metric for instantaneous WSS vector components that are perpendicular to the mean WSS vector in the local endothelium plane, as proposed previously.^40^

### Statistical Analysis

Data are expressed as mean±SEM and were analyzed using Prism software (v6; GraphPad). Paired blood pressure data were analyzed by paired 2‐way ANOVA followed by Sidak posttest. For 2‐group comparisons, data were analyzed by Student *t* test for normally distributed data. Statistical significance was achieved when *P*<0.05.

## Results

**Figure 1:**
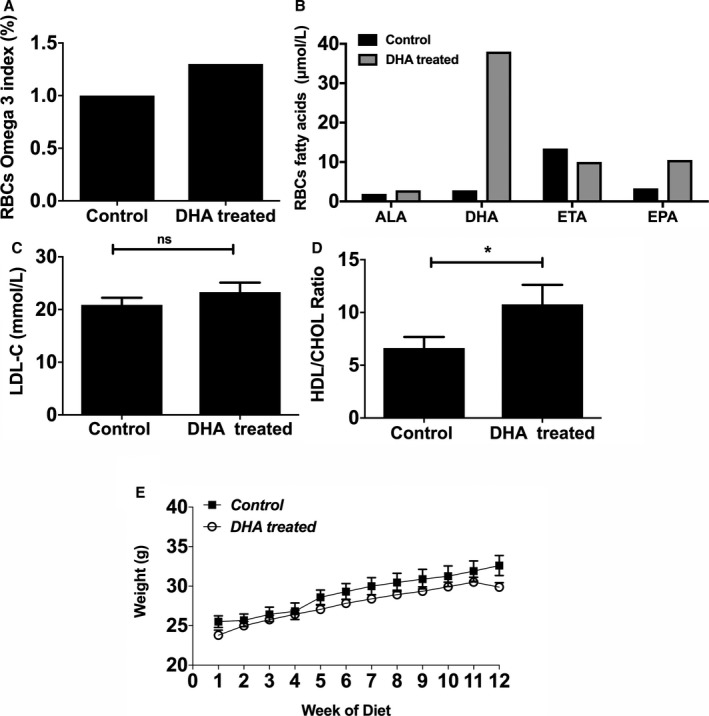
DHA feeding of apoE^−/−^ (apolipoprotein E–null) mice induces changes in RBC ω‐3 fatty acid compositions without any significant effect on their body weights. A, The ω‐3 index (percentage) is enhanced in DHA‐fed mice compared with controls. The apoE^−/−^ mice were fed an HFD alone (control) or an HFD and DHA (300 mg/kg per day) for 12 weeks. Data are from pooled blood of n=4 mice per group. Blood was sampled at the experimental end point. B, Fatty acid composition in RBCs of DHA‐fed mice compared with controls (μmol/L; pooled blood from 4 mice per group). C, Plasma LDL‐C (in mmol/L) and (D) HDL/CHOL ratio, measured enzymatically (n=10 per group). E, Body weights of the mice (in g) were recorded weekly. Data are shown as mean±SEM, analyzed by Student *t* test (C and D) or 2‐way ANOVA followed by Tukey test (E), *P<0.05. ALA indicates α‐linolenic acid (18:3n‐3); DHA, docosahexaenoic acid (22:6n‐3); EPA, eicosapentaenoic acid (20:5n‐3); ETA, eicosatetraenoic acid (20:4n‐3); HDL/CHOL, high‐density lipoprotein/total cholesterol; HFD, high‐fat diet; LDL‐C, low‐density lipoprotein cholesterol; RBC, red blood cell.

DHA supplementation of the HFD was well tolerated by the mice, with no side effects, and absorption was confirmed by specific changes in free fatty acid composition (increased by 38 μmol/L in DHA‐fed mice) in red blood cell membranes (Figure 1B). The red blood cell ω‐3 index in the DHA‐fed mice increased by an average of 25% compared with the control mice (anecdotal observation under experimental conditions; Figure 1A).

Plasma levels of LDL‐C (low‐density lipoprotein cholesterol) in DHA‐treated mice were not significantly different from those in the controls (Figure 1C), but the HDL (high‐density lipoprotein)/cholesterol ratio significantly increased in the DHA‐supplemented mice compared with controls (Figure 1D). There was no significant change in body weight between the 2 groups (Figure 1E).

**Figure 2:**
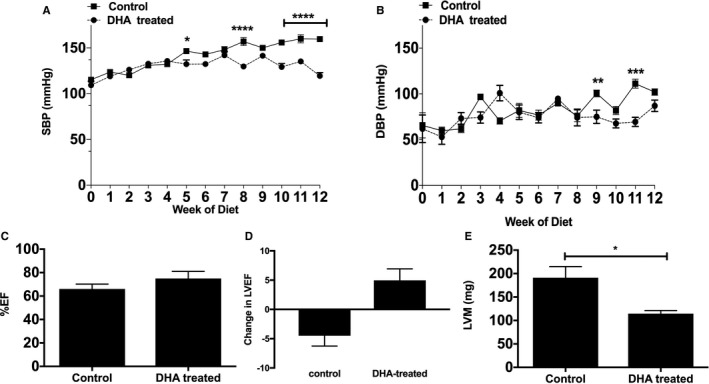
DHA significantly reduces HFD induced hypertension in apoE^−/−^ (apolipoprotein E–null) mice. A, SBP and (B) DBP were measured in freely moving apoE^−/−^ mice fed either HFD alone (control) or HFD and DHA (DHA treated) for 12 weeks (n=4 per group) using a tail‐cuff system (see Methods for details). All data are expressed as mean±SEM, analyzed by 2‐way ANOVA and Tukey posttest, *P<0.05, **P<0.01, ***P<0.001, ****P<0.0001. Effects of DHA feeding on (C) left ventricular EF (%EF) and change in (D) EF and (E) LVM (in mg). Echocardiographic data were measured in anesthetized mice and are presented as mean±SEM (n=4 per group), analyzed by unpaired Student *t* test, *P<0.05. DBP, diastolic blood pressure; DHA, docosahexaenoic acid (22:6n‐3); EF, ejection fraction; HFD, high‐fat diet; LVM, left ventricular mass; SBP, systolic blood pressure.

We analyzed blood pressure data collected over the feeding period. At baseline, there was no difference in starting values between groups. Subsequently, with feeding, systolic blood pressure rose in both groups until week 5 and then declined in the DHA‐supplemented group only (132.3±9.18 versus 146.6±3.563 mm Hg, respectively, *P*<0.05, n=4). This fall in systolic blood pressure in the DHA‐supplemented group continued for a further 7 weeks (119.5±7.33 mm Hg in the DHA group compared with 159.7±2.482 mm Hg, *P*<0.001 at week 12; Figure 2A). Diastolic blood pressure in the DHA‐fed mice also significantly decreased compared with the control group (75±14.65 versus 100.5±7.549, respectively; *P*<0.01; Figure 2B).

Changes in LV wall thickness and function were recorded using echocardiography and were analyzed at week 0 (baseline) and week 12. In the DHA‐supplemented group, the percentage of LV ejection fraction increased after 12 weeks, although this was not statistically significant compared with control mice (Figure 2C). However, the change in ejection fraction between time 0 and 12 weeks showed the control group experienced a significant decrease, whereas the DHA group had an increase in ejection fraction (Figure 2D). LV mass was significantly decreased in the DHA group after 12 weeks (Figure 2E) compared with controls. Collectively, these data suggest that DHA supplementation may protect against LV hypertrophy induced by HFD feeding in apoE^−/−^ mice, secondary to high blood pressure.

**Figure 3:**
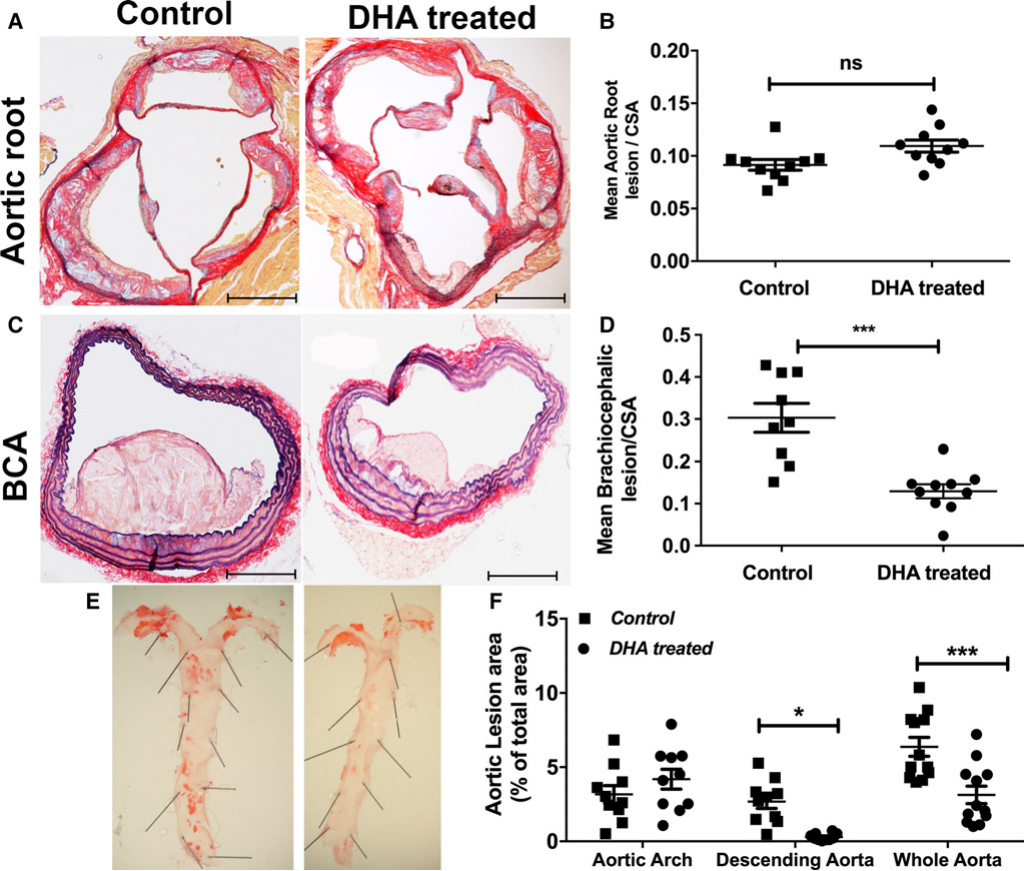
Differential effects of DHA feeding on lesion development in different areas of the vascular beds. Male apoE^−/−^ (apolipoprotein E–null) mice aged 8 weeks were fed a Western‐type HFD alone (control) or an HFD and jelly containing DHA (DHA treated; 300 mg/kg per day) daily over 12 weeks. A, Representative images of aortic roots stained with AB/EVG after 12 weeks of feeding (n=12 per group). Scale bars=100 μm. B, Mean lesion area of aortic root sections, normalized to CSA (n=12). Data are mean±SEM, analyzed by unpaired Student *t* test, *P*=ns. C, Representative images of brachiocephalic sections stained with AB/EVG (scale bars=100 μm). D, Mean lesion area of brachiocephalic arteries, normalized to CSA. Data are mean±SEM, analyzed by unpaired Student *t* test, 9 or 10 per group, ***P<0.001. E, Representative en face morphometric images of the total aortic lesion area and (F) whole aortic, aortic arch, and descending aortic lesion area calculated as a percentage of the total surface area of the whole aorta, showing significant reduction in the total lesion formation in the distal aorta in the DHA group compared with control. Data are mean±SEM, analyzed by 2‐way ANOVA and Tukey post‐test, 11 or 12 per group, *P<0.05, ***P<0.001. AB/EVG indicates alcian blue and elastic van Gieson; CSA, cross‐sectional area; DHA, docosahexaenoic acid (22:6n‐3); HFD, high‐fat diet.

Atherosclerotic lesion analysis showed no difference in lesion size in the aortic root (Figure 3A and 3B) but a significant reduction in lesion size in brachiocephalic artery in DHA‐supplemented HFD mice versus controls (Figure 3C and 3D).

The distribution of plaques in the whole thoracic aorta was significantly altered with DHA feeding, leading to a 45±12% decrease in the total aortic Oil Red O–stained atherosclerotic area (*P*<0.001) compared with controls (Figure 3E and 3F). This effect was localized in the descending aortic plaques only (a reduction of 30±5%, *P*<0.05; Figure 3F), with no effect of DHA supplementation observed in the aortic arch.

**Figure 4:**
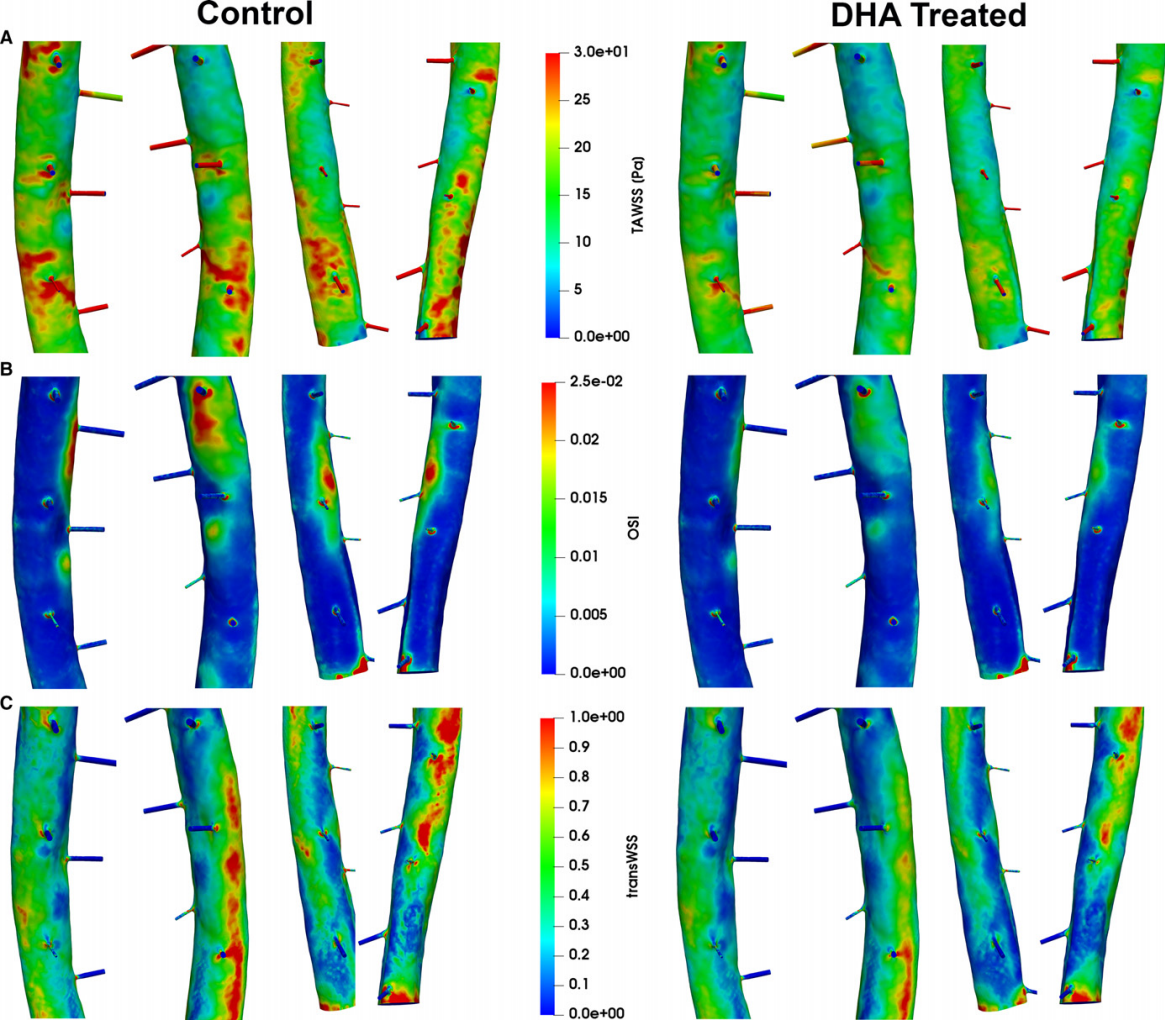
Flow simulations for descending aortas with intercostal branches dissected from DHA‐treated and non–DHA‐treated pro‐atherosclerotic mice. Shown are (A) TAWSS magnitude (TAWSS), (B) OSI, and (C) transWSS for left upper, right upper, left lower, and right lower 3 pairs of the ostia (from left to right) for the control group vs the DHA‐treated group. Flow characteristics remain similar between control and DHA‐treated vessels but show elevated wall shear stress levels throughout that correlate with the higher maximum flow speed. OSI shows only a small decrease in DHA‐treated animals, located mainly below and between ostia branches. TransWSS is again higher in the control group. DHA indicates docosahexaenoic acid (22:6n‐3); OSI, oscillatory shear index; TAWSS, time‐average wall shear stress; transWSS, transverse wall shear stress.

Optical projection tomography imaging of the descending aorta and its intercostal vessels and reconstruction of slices to make a 3‐dimensional macrogeometry were used to build a computational fluid dynamic model of blood flow at intercostal branches. Using flow parameters from our echocardiography study and estimated boundary conditions from ourselves^34^ and others,^41^ simulations of flow showed a reduction in time‐averaged WSS (23.63±1.9 versus 18.53±1.5 Pa, *P*<0.001) and, more important, both OSI (0.004±0.001 versus 0.003±0.0002, *P*<0.01) and transWSS (0.355±0.035 versus 0.285±0.03 Pa, *P*<0.001) in mice supplemented with DHA (Figure 4).

**Figure 5:**
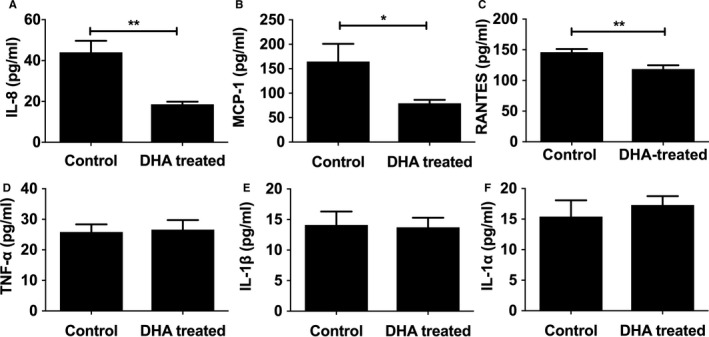
Plasma pro‐inflammatory profiles in response to DHA feeding. Male apoE^−/−^ (apolipoprotein E–null) mice, from 8 weeks of age, were fed either an HFD alone or an HFD and DHA (300 mg/kg per day) daily. Following 12 weeks of diet, freshly isolated plasma was analyzed using cytometric bead arrays for (A) IL‐8, (B) MCP‐1, (C) RANTES, (D) TNF‐α, (E) IL‐1β, and (F) IL‐1α (in pg/mL; 8–10 per group). Data are expressed as mean±SEM, analyzed by unpaired Student *t* test, *P<0.05, **P<0.01. DHA indicates docosahexaenoic acid (22:6n‐3); HFD, high‐fat diet; IL, interleukin; MCP‐1, monocyte chemoattractant protein 1; TNF‐α, tumor necrosis factor α.

**Figure 6:**
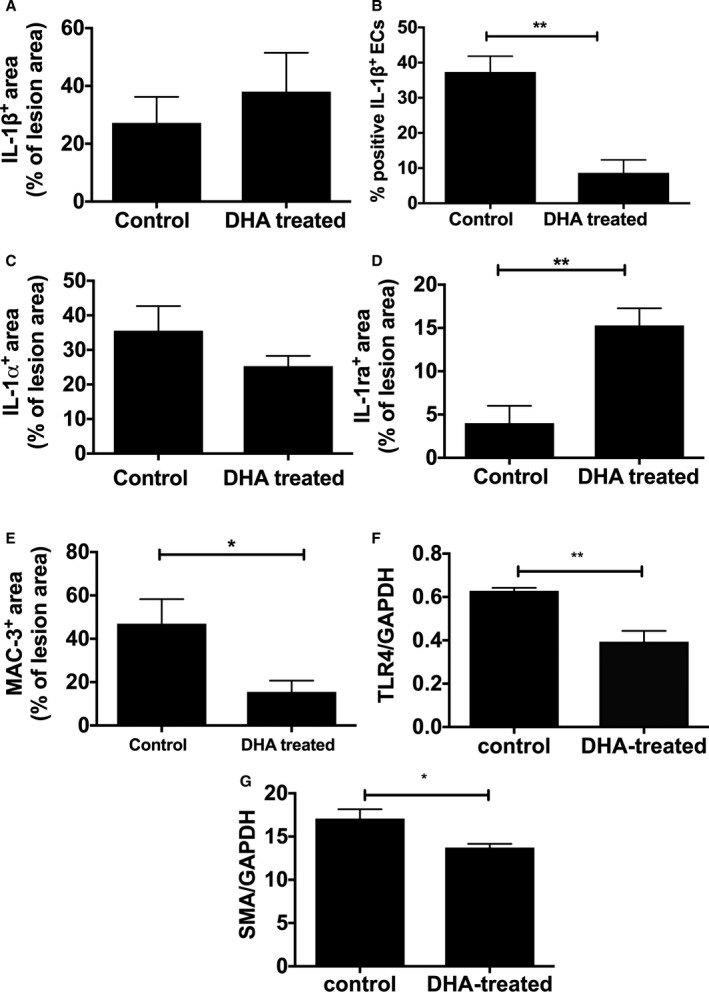
IL‐1 distribution in aortic atherosclerosis and lesion characteristics in response to DHA feeding. Male apoE^−/−^ (apolipoprotein E–null) mice, from 8 weeks of age, were fed either an HFD alone or an HFD and DHA (300 mg/kg per day) daily. Following 12 weeks of diet, immunostaining, measured semiquantitatively as a percentage of total lesions, showed no difference in (A) IL‐1β or (B) IL‐1α. The number of IL‐1β–positive ECs, measured semiquantitatively as a percentage of total number of ECs, is lower in DHA‐treated mice compared with control (B). D, IL‐1ra is increased and (E) Mac‐3 (CD107b) is decreased in DHA‐treated animals. Image analysis was performed using NIS‐Elements software, and data are represented as mean±SEM, 6 to 8 per group. Student *t* tests indicate a significant difference with *P<0.05; **P<0.01. Western blot analysis of mouse aortas showed (F) TLR‐4 and (G) SMA levels are significantly decreased following DHA feeding. DHA indicates docosahexaenoic acid (22:6n‐3); EC, endothelial cell; HFD, high‐fat diet; IL, interleukin; SMA, smooth muscle actin; TLR‐4, Toll‐like receptor 4.

**Figure 7:**
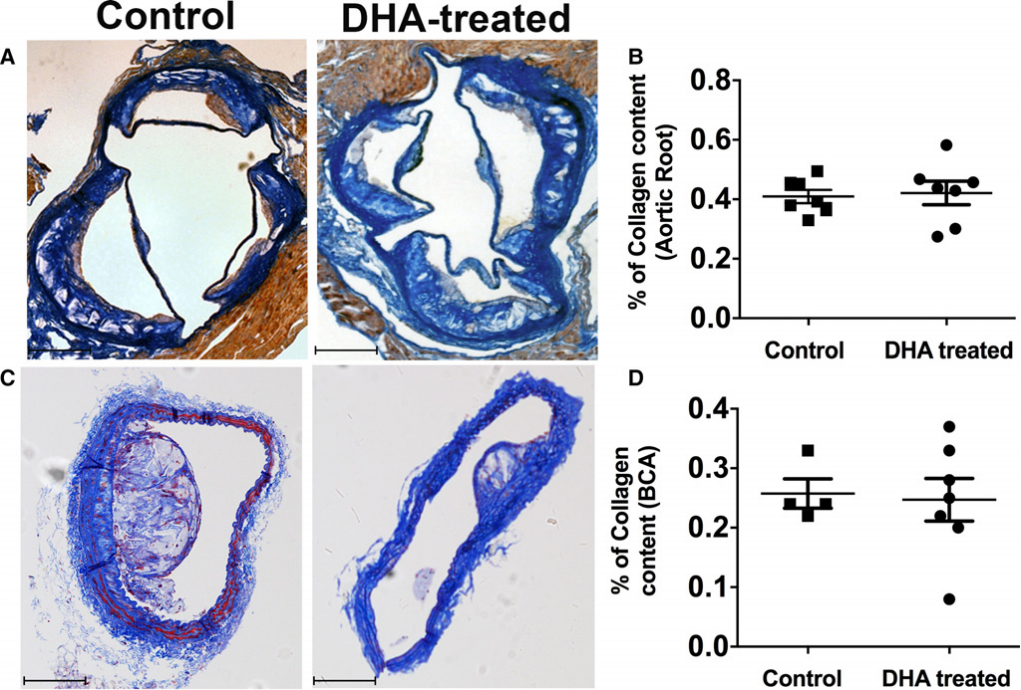
Collagen content in the different vasculature is not affected by DHA feeding. A, Collagen content in the aortic root of studied groups. Representative images of aortic roots of apoE^−/−^ (apolipoprotein E–null) mice fed with an HFD alone or an HFD and DHA for 12 weeks, stained for martius scarlet blue. Collagen stains bright blue. Scale bars=100 μm. B, Quantification of the collagen content within the aortic roots of the 2 studied groups, measured as a percentage of the total lesion area. C, Collagen content in BCAs. Representative images of BCA of apoE^−/−^ mice fed with an HFD alone or an HFD and DHA for 12 weeks, stained for martius scarlet blue. Collagen stains bright blue. Scale bars=100 μm. D, Quantification of the collagen content within the BCAs of the 2 studied groups measured as a percentage of the total lesion area. Data are shown as mean±SEM, n=7 per group, analyzed by unpaired Student *t* test, *P*=ns. BCA indicates brachiocephalic artery; DHA, docosahexaenoic acid (22:6n‐3); HFD, high‐fat diet.

Analysis of inflammatory cytokines showed mice fed a diet supplemented with DHA had reduced plasma IL‐8, regulated on activation, normal T cell expressed and secreted; CCL5 (RANTES), and MCP‐1 (monocyte chemoattractant protein 1), but there was no change in TNF‐α (tumor necrosis factor α) or IL‐1 versus controls (Figure 5A–5F); however, we were able to measure the apical cytokine IL‐1β directly in atherosclerotic lesions by immunostaining. Although we saw no difference in IL‐1β levels in the lesion as a whole (Figure 6A), we did observe a significant reduction in endothelial IL‐1β expression in the DHA‐supplemented mice compared with controls (Figure 6B). IL‐1α levels did not differ between groups (Figure 6C). Levels of IL‐1ra, however, were increased in DHA‐fed mice (Figure 6D). Concomitantly, Western blot analysis of aortas showed levels of TLR‐4 (Toll‐like receptor 4) were reduced following DHA feeding (Figure 6F). Analysis of aortic sections for macrophages showed a significant reduction in DHA‐treated animals compared with controls (Figure 6E). There were no differences in collagen levels (Figure 7) between groups; however, there was a reduction in smooth muscle cells, analyzed by Western blotting for smooth muscle actin, in DHA‐fed mice (Figure 6G).

## Discussion

Contemporary primary and secondary therapies for cardiovascular disease prevent the progression of atherosclerosis and stabilize the plaque, providing significant clinical benefit^42^; however, not all patients respond to risk factor–modifying therapy, especially patients with hypertension.^43^, ^44^, ^45^ The Ω‐3 fatty acids, particularly DHA (a major type of n3FA in fish oil) and α‐linolenic acid (ALA; the most common land‐based type of n3FAs), have been shown to play an important role in preventive medicine.^46^, ^47^ A diet supplemented with n3FAs leads to increased incorporation of the n3FAs (ALA, DHA, eicosapentaenoic acid) in plasma lipoproteins^48^ associated with lower rates of plaque vulnerability.^49^, ^50^ In clinical studies, however, DHA has been mostly mixed with eicosapentaenoic acid or given as fish oil, and thus its individual effects remain obscured. ALA may have a cardioprotective role; however, the overall evidence is mixed and remains inconclusive.^51^, ^52^ Similarly, several animal models of atherosclerosis have investigated the anti‐atherogenic effects of n3FAs,^53^ but these studies also did not directly define the differential effects of individual n3FAs on atherosclerosis. To our knowledge, this study is the first to define a common anti‐atherogenic and anti‐hypertensive mechanism of DHA. It is also the first to perform fluid dynamics analysis of blood flow around the intercostal branches in a mouse and to suggest a potential mechanism by which DHA alters flow patterns in the distal aortas, leading to modulation of inflammation.

It is published that n3FAs have a blood pressure–lowering effect in both normotensive^54^, ^55^ and hypertensive^56^ individuals. In addition, studies using animal models have shown that fish oil has a generalized blood pressure–lowering effect.^57^ Nevertheless, as with atherosclerosis, the individual effects of n3FAs in hypertension are less studied. DHA is more commonly investigated for its anti‐hypertensive effects,^27^ and there is an endogenous conversion for DHA to eicosapentaenoic acid occurring mainly in the liver.^58^ Consequently, this study focused on the specific effects of DHA on atherosclerosis‐related hypertension.

Our data show that DHA significantly decreases blood pressure in our experimental model, reinforcing previous interventional studies in humans^59^, ^60^ and consistent with a report that DHA supplementation significantly reduces 24‐hour ambulatory blood pressure in hypercholesterolemic patients.^61^ In animal studies, DHA reduces systolic blood pressure and vascular wall thickness in spontaneously hypertensive rats^25^, ^62^ and aldosterone‐induced hypertension in dogs.^27^ Our study, however, is the first to elucidate the hypotensive effects of DHA in experimental atherosclerosis induced in mice by feeding a Western diet.

In contrast, ALA supplementation did not show any significant impact on blood pressure in atherosclerotic mice (Mabruka A Alfaidi, PhD, 2017, unpublished data). ALA as an antihypertensive agent has been studied in a limited number of studies,^63^ and the lack of effect on blood pressure with ALA is at odds with previous clinical studies that demonstrate the hypotensive effects of ALA in hypertensive individuals.^56^ Mice, however, are deficient in the enzyme that is responsible for the endogenous metabolism of ALA^64^ and thus may respond differently to ALA than humans. As such, ALA investigations were not pursued in this study.

An independent association exists between LV mass and atherosclerotic coronary disease in patients with IHD,^65^ and we observed that supplementation with DHA significantly attenuated the increase in LV mass after 12 weeks of HFD feeding in mice. Our finding of a significant reduction of total aortic tree lesions by DHA is in agreement with another recent study.^66^ However, we go further than this and show that DHA supplementation of a Western diet in mice significantly and selectively decreases atherosclerosis in the distal parts of the aorta and brachiocephalic arteries. Such differential atherosclerotic responses have been reported before but have not been explained.^67^ Our novel computational flow analyses indicate that flow effects are part of the explanation for this, and we postulate that the effect of DHA is more pronounced in areas with a high oscillatory or a high transverse component of the WSS compared with areas where there is high average WSS. In some locations along the aorta (especially in the arch), the plaque formation in the control group correlates with high OSI, whereas in regions with generally low OSI, there seems to be a correlation with high transWSS. The exact relationship between these WSS derivatives and atherosclerosis is not yet fully understood, although hypertensive remodeling of resistance vessels is postulated to be due to a combination of genetic factors, maladaptation of walls of vessels in the microcirculation to adverse mechanical conditions, and the influence of neurohumoral and local trophic factors.^68^, ^69^, ^70^

The local anti‐inflammatory effect of DHA has been previously associated with a thickening in the fibrous cap and more stable plaque formation.^45^ In our study, however, smooth muscle cell content was decreased and collagen content was not altered. DHA is known to suppress smooth muscle cell proliferation in cell culture,^20^, ^71^ and our data suggest that the mechanism of atheroprotection mediated by DHA may be complex, linked to blood flow patterns and WSS affecting inflammation locally and at distal atheroprone sites.

We suggest that DHA, through modulation of complex blood flow potentially via changes in viscosity,^72^ transduces and inhibits apical inflammatory signals that orchestrate inflammation in atherosclerosis. Human studies suggest that supplementing the diet of young males with DHA in fish oil significantly reduces IL‐1β production in LPS (lipopolysaccharide)‐stimulated monocytes.^73^ In addition, Vijay‐Kumar et al have reported that monocytes harvested from mice fed fish oil containing DHA produced less IL‐1β compared with controls.^74^ We found, however, that DHA supplementation in mice had no effect on the plasma levels of IL‐1. The discrepancy between our data and these published results could occur because measurement of cytokines in plasma is notoriously difficult or because, in our model, DHA may decrease local production of cytokines such as IL‐1β in cells inside lesions themselves. Indeed, we observed a decrease in endothelial IL‐1β expression in atherosclerotic lesions of DHA‐fed animals versus controls. The primary cellular origin of IL‐1β in atherosclerosis is unclear, but we have previously shown in coronary atherosclerotic plaques of patients with IHD that IL‐1β is predominantly expressed in relatively large amounts in the endothelium and vasa vasorum.^75^ We did show robust reductions in IL‐8, RANTES, and MCP‐1 in plasma, although previous animal studies have reported no significant correlation between DHA feeding and these plasma cytokines.^76^ Most of these studies, however, were small and used fish oil, with little attention given to specific DHA effects. Our findings do agree with data from in vitro studies, particularly from cultured cells, including monocytes and endothelial cells.^77^, ^78^, ^79^, ^80^ We thus conclude that the atheroprotective effect of DHA is transduced by alterations in flow leading to an arterial environment in which levels of these plasma chemoattractants are reduced.

**Figure 8:**
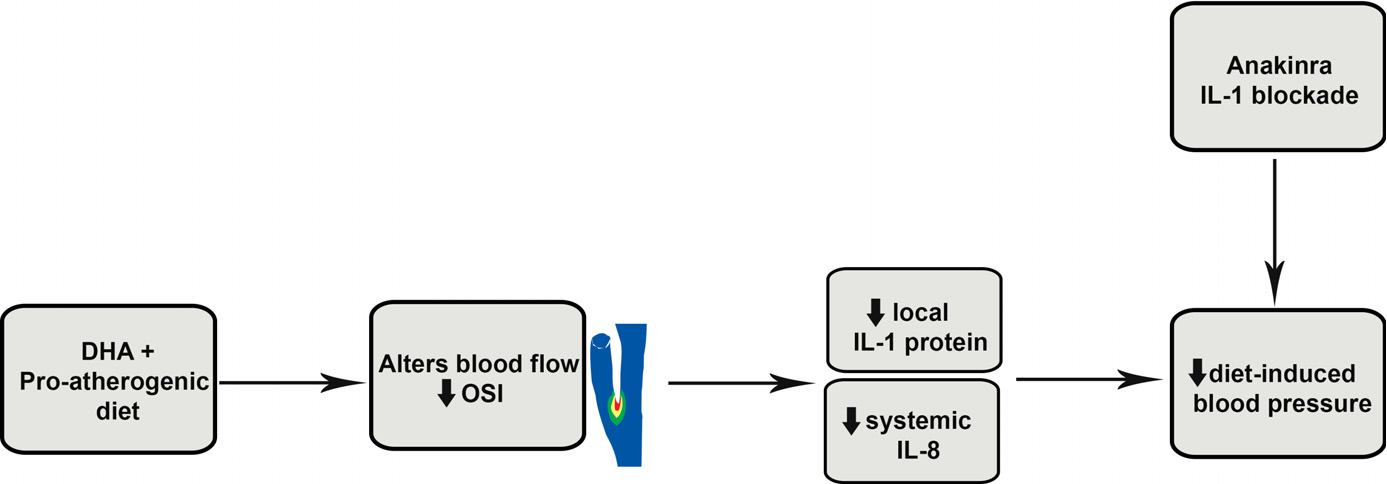
Schematic showing the potential mechanism whereby DHA selectively leads to alterations in oscillatory shear in distal vessels, reduced inflammation, and lower blood pressure. DHA indicates docosahexaenoic acid (22:6n‐3); IL, interleukin; OSI, oscillatory shear index.

Our previous studies have shown that atherosclerotic mice treated with anakinra or the murine version of canakinumab, as well as IL‐1R1 knockout mice, have reduced blood pressure and atherosclerosis formation compared with controls^9^, ^81^ and that this finding is replicated in our DHA‐fed mice. The reduction in IL‐1‐induced biomarkers (IL‐8, MCP‐1, RANTES) in mice fed DHA is also replicated in these mice when IL‐1 signaling is inhibited. This combined with a reduction in TLR‐4 levels and an increase in IL‐1ra levels in DHA‐fed mice all suggest DHA is acting via an IL‐1 mechanism (Figure 8). Furthermore, and of potential clinical relevance, unpublished data from the IL‐A HEART study^82^, ^83^ in our group show that in a post hoc secondary analysis of blood pressure data, systolic and diastolic blood pressure and mean arterial pressure decreased in the group receiving IL‐1ra (baseline versus day 14: 131.4±2.4 versus 124.7±1.6 mm Hg, systolic, *P*<0.01; 75.2±1.5 versus 71.1±1.2 mm Hg, diastolic, *P*=ns; 93.9±1.7 versus 88.6±1.2 mm Hg, mean arterial pressure, *P*<0.05; n=76 patients [IL‐1ra] and n=71 [placebo], *t* test). Given other recent data,^84^ we speculate that strategies to block IL‐1 signaling directly could modulate hypertension.

Our DHA study does have limitations. It is not clear, for example, whether the effect on atherosclerosis is related to a direct effect of DHA on blood pressure or whether the opposite applies. The same limitation applies to our previous studies that lowered blood pressure by inhibiting IL‐1 signaling directly, without using DHA. To fully address this question, analysis of atherosclerosis in a study group whereby blood pressure is lowered by a non‐DHA and non–IL‐1 mechanism would need to be performed.

In conclusion, our study provides the first evidence that regular supplementation of DHA in the proatherogenic diet of mice influences arterial walls in such a way as to alter blood flow patterns distally in the vascular tree. We suggest that this may lead to reduced local inflammation (IL‐1 production) in vessel walls; reduced lesion burden in distal vessels; and, consequently, lowered blood pressure. Further studies targeting IL‐1 for lowering hypertension directly in mice or humans are warranted.

## Sources of Funding

This work was supported by a PhD studentship grant to Dr Alfaidi, from Medical School, Omar Almokhtar University, Al‐Bayda, Libya, and by British Heart Foundation grant PG/13/8/29989, UK Medical Research Council Experimental Medicine Grant (G0502131), the National Institute for Health Research, and a UK Medical Research Council Clinical Research Training Fellowship (AR‐MR/K002406/1). Rothman is supported by a Wellcome Trust Clinical Research Career Development Fellowship (206632/Z/17/Z).

## Disclosures

None.

## Supporting information


**Data S1.** Supplemental methods.
