# Supplementary material for: Dietary Docosahexaenoic Acid Reduces Oscillatory Wall Shear Stress, Atherosclerosis, and Hypertension, Most Likely Mediated via an IL‐1–Mediated Mechanism
Source: J Am Heart Assoc. 2018 Jun 30;7(13):e008757. doi: 10.1161/JAHA.118.008757 (PMC6064924; doi:10.1161/JAHA.118.008757)
Supplement: Supplementary file 1 — Data S1. Supplemental methods. [file JAH3-7-e008757-s001.pdf]

SUPPLEMENTAL MATERIAL

Data S1.

Supplemental Methods

Animals and Diets

Male ApoE$^{-/-}$ mice were bred in-house at the University of Sheffield. Food and water were given *ad libitum* under a controlled environment (Temp. 22-25°C, humidity 55±5 and 12h light cycle). Mice were housed individually from 8 weeks of age and randomly separated into one of two groups (n=12/group). The control group was fed a high fat, Western-type diet (HFD) containing 21% (w/w) fat, 0.15% (w/w) cholesterol and 0.296% (w/w) sodium (Special Diet Services, Witham, UK) whereas the DHA treated group received HFD and DHA (99% purified, Sigma-Aldrich, UK). To ensure that the mice received the same concentration of DHA on each individual day, the free fatty acid was mixed with jelly at equal and final concentrations of 300mg/kg/day, and the mice were monitored daily for their fatty acid (jelly) intake. The method of using jelly to deliver drugs to mice was published recently by (30) and has shown to be an efficient way to deliver drugs to mice at the required concentrations. The mice were trained for at least one week with the jelly alone, and then the jelly containing DHA was given to the mice along with the HFD.
All animal care and procedures were closely conducted under ASPA 1986.

Blood pressure analysis

Systolic and diastolic blood pressure was measured in the mice using a Visitech tail-cuff system (Visitech Systems, NJ, USA) as described previously (32). Briefly, the mice were subjected to one week of training before starting the actual measurements in order to minimize stress levels. Blood pressure was measured in 4 mice per group, and 10 measurements per mouse per day were recorded for four different days per week for 12 weeks. The data were rejected if the systolic blood pressure was more than 200mmHg or less than 40mmHg, outside the 2SD of the mean or had fewer than 4 valid readings. Per week measurements were also rejected if they had fewer than 3 valid readings.

Echocardiography

To assess the effect of DHA feeding on cardiac function during the duration of the study, transthoracic echocardiogram (TE) was performed as previously described (33). Briefly, the mice (n=4/group) were anesthetised using isoflurane delivered in oxygen in an isolator before placing the mice in a supine position. To minimise excessive heat loss, which may affect the measurements, the mice were placed on a heated platform and continuous rectal temperature was monitored.
During recording, the mice were maintained on approximately 0.5-1.5% (v/v) isoflurane in oxygen, delivered via a nose cone. The level of anesthesia was adjusted to achieve a heart rate close to 500 ± 125bpm. To allow smooth image acquisition, the mouse chest wall was depilated and ultrasound gel was applied (Aquasonics 100 Gel, Parker Labs Inc. New Jersey, U.S).
Left ventricular (LV) function was recorded in the short axis view at the level of the mid-papillary muscles, whereas M-mode measurements were made for LV wall and cavity dimension (LVIDd). Ejection fraction (%EF), fractional shortening (%FS) and corrected LV mass (LVM) were assessed using automated analysis.

Plasma profiles

The blood was collected using cardiac puncture and plasma separated by centrifugation at 3000g over 5 minutes, collected and stored at -80°C prior to analysis. The inflammatory markers (IL-6, IL-8, RANTES, and MCP) were measured in the plasma using a cytometric bead array (CBA; BD Bioscience) according to manufacturer's recommendations. Lipid profiles (total cholesterol, LDL and HDL/cholesterol ratio) were measured in the Clinical Biochemistry Laboratory at the Royal Hallamshire Hospital, Sheffield, UK.

Preparation of samples for erythrocyte fatty acid analysis
Freshly collected blood (by cardiac puncture) was collected into 4ml EDTA tubes (4 pooled samples of RBCs from 4 different mice per group with a minimum volume of 1mL). The blood samples were centrifuged for 5 minutes at 3000g and the plasma and buffy coat were aspirated and discarded. The tubes containing red cell fractions were then labelled and stored at -80^{0}C until sent to BioLab, London, UK to conduct the analysis.

Atherosclerosis analysis
The extent of atherosclerosis was assessed in whole aortae by an *en face* method (35). In brief, the aortae were perfused firstly by PBS and then by 10% (v/v) formalin. After the exposure of the whole aortae and removal of the adherent fats and vessels, aortae were dissected from the levels of aortic valves to the aortic orifice of the diaphragm under a dissecting microscope. Aortae were then fixed in 10% (v/v) formalin overnight at 4°C and stored in PBS at 4°C until pinning was conducted.
The aortae were opened longitudinally and stained with oil red O stain (ORS). For this, the aortae were rinsed in 60% (v/v) isopropanol and stained with 0.3% (w/v) Oil Red O (Sudan IV; Sigma O0625) in PBS for 30min followed by destaining for 20min in 60% (v/v) isopropanol and further washing in distilled water. The stained aortae were pinned on a wax filled petri dish (15cm) using micro-needles (Fine Science Tools, Heidelberg, Germany). Images were recorded using a digital camera connected to light microscope at 15x magnification. Lesion areas were analysed using a NIS-elements analysis software system (Nikon, UK). Atherosclerotic lesion in the whole aortae, arch and descending parts were quantified as % of the total surface area.
A second assessment of atherosclerosis was conducted in cross-sectional aortic root and brachiocephalic sections as described (34). Briefly, the hearts and brachiocephalic arteries (first branch of the aortic arch) were collected and stored in 10% (v/v) formalin overnight at 4°C and then in PBS until they were embedded in paraffin wax. The paraffin-embedded hearts (at the level of aortic valves) and brachiocephalic arteries were serially sectioned using a Leica RM2135 microtome (Leica Microsystems, Wetzlar, Germany). Sections (5μm thickness) were collected and stained with Alcian Blue & Elastic Van Gieson (AB/EVG) as described (30).

Analysis of Collagen content
Collagen content in aortic and brachiocephalic sections were measured in martius scarlet blue (MSB) positive stained areas as described (30) and expressed as a percentage of the total surface area. In brief, the tissues were dewaxed and rehydrated in xylene and graded alcohols. The tissues were then stained with 1% (w/v) Celestine blue for 5 minutes, drained, and stained with Harris' haematoxylin for 5 minutes.

After rinsing in water and differentiating in acid alcohol for few seconds, the tissues were exposed to hot running water for 5 minutes and rinsed in 95% (v/v) ethanol and then stained with martius yellow (0.5% (w/v) martius yellow, 2% (w/v) phosphotungstic acid in 95% (v/v) ethanol) for 2 minutes.

The sections were then stained with Ponceau de xylene (1% (w/v) ponceau de xylene in 2% (v/v) glacial acetic acid) for 10 minutes and differentiated in 1% (w/v) phosphotungstic acid for 5 minutes. After draining of the stained sections, the tissues were stained with methyl blue (5% (w/v) methyl blue in 10% (v/v) glacial acetic acid) for 10 minutes and rinsed in 1% (v/v) acetic acid for 10 minutes. The sections were then dehydrated through graded alcohols and xylene and mounted using DPX mounting media. Analysis was performed using NIS-Element software.
Alcohols and xylene were purchased from Thermo Fisher Scientific whereas all stains and DPX resin were obtained from VWR International Ltd (Lutterworth, UK) unless otherwise stated.

Statistical analysis
Data are expressed as mean ± SEM and analysed using prism software (Version 6, GraphPad, San Diego, CA). Blood pressure data were analysed by 2-way ANOVA followed by Tukey’s post-test. For the two-group comparison, data were analysed by unpaired Student *t* test for normally distributed data. Statistical significance was achieved when $p<0.05$.
